# Thidiazuron and *trans*-zeatin riboside promote development of shoots and nodes in *in vitro* propagation of hops (*Humulus lupulus* L. var. *lupulus*)

**DOI:** 10.3389/fpls.2025.1694038

**Published:** 2026-01-13

**Authors:** Elise R. Staats, Maria M. Jenderek

**Affiliations:** United State Department of Agriculture (USDA), Agricultural Research Service (ARS), Agricultural Genetic Resources Preservation Research Unit, Fort Collins, CO, United States

**Keywords:** double magenta vessels, hyperhydricity, main shoot length, micropropagation, nodes, plant growth regulators, shoots

## Abstract

Hops (*Humulus lupulus* L. var. *lupulus*) are dioecious, climbing plants considered as a horticultural and industrial crop. They are propagated vegetatively by root cuttings and, to a lesser extent, by tissue cultures. Various plant growth regulators were applied in hops’ tissue culture propagation, such as benzyladenine, kinetin, indole-3-acetic acid, thidiazuron, zeatin, and gibberellic acid, and some in combination with glucose. *In vitro* propagation of three randomly selected genotypes (PI 546055, PI 558687, and PI 617389) from the US Department of Agriculture (USDA) Agricultural Research Service (ARS) hop germplasm collection was carried out to establish a procedure for developing a large quantity of shoots, nodes, and long main shoots using 15 treatments of plant growth regulators and glucose without plant growth regulators. The micropropagation was carried out in three propagation cycles lasting 4 weeks each. The largest number of shoots and nodes resulted from treatments with thidiazuron (15.9 ± 1.34 shoots; 40.6 ± 3.6 nodes). Literature has reported the successful use of this plant growth regulator in selected hop cultivars, but thidiazuron caused hyperhydricity in PI 546055. The occurrence of hyperhydricity in this accession seems to be genotype-dependent. Hence, its use in hop micropropagation should be controlled. The disorder was not observed in the other treatments. In the 3.0 mg L^−1^ of *trans*-zeatin riboside treatment, the shoot number (8.4 ± 1.34) and node number (24.9 ± 3.6) were not much different from those of the thidiazuron treatments, and the length of the main shoots (9.3 ± 0.92 cm) was not significantly different from that of the other treatments, except for the thidiazuron treatments. The 3.0 mg L^−1^ of *trans*-zeatin riboside was the top treatment for the micropropagation of the three hop accessions. This treatment will be used for a variety of accessions from the USDA hop germplasm collection. It may also be useful when large quantities of plantlets are needed for other purposes and when the responsiveness to thidiazuron (TDZ) is unknown.

## Introduction

Hops (*Humulus lupulus* L. (Cannabaceae) are an industrial and horticultural crop mainly used in beer production and herbal medicine. In 2024, its global market value was $9.4 billion (IMARC). The two largest hop producers are the United States and Germany (Kubeš, 2022). In recent years, its cultivation acreage has decreased, but due to increased yields, production has marginally increased. Hops are a dioecious, perennial plant, and only female plants produce cone-shaped flowers, which are cultivated in fields and utilized (Iacuzzi et al., 2023; Lagos et al., 2022). The crop is propagated vegetatively by cuttings of stems, rhizomes (MaChado et al., 2018), and tissue culture; however, its last multiplication method is not frequently used. According to Hirakawa and Tanno (2022), protocols for *in vitro* propagation of hops are needed. Tissue culture propagation supports not only large-scale plant preparation for planting but also other aspects of biological processes, including germplasm preservation (Gamborg et al., 1976; Reed et al., 2003). Various plant growth regulators (PGRs) were used in hop micropropagation. In some cultivars, axillary bud development was induced by 0.01–0.05 ppm of gibberellic acid (GA_3_) combined with a low concentration of benzyladenine (BA). High concentrations of BA blocked the induction of axillary buds (Hirakawa and Tanno, 2022). In some species of the Cannabaceae family, cytokinins promoted the development of axillary buds but at the same time repressed the elongation of the main shoot. The length of the main shoot depended on the PGR concentration (Bychkova et al., 2024). The same authors reported the longest main shoot in an ‘AK-3’ wild accession cultivated on Murashige and Skoog (MS; 1962) medium with 5 μM of kinetin and in a variety ‘Civil’skij’ also on MS medium with 1 μM of BA. Mafakheri and Hamidoghli (2019) reported the highest number of shoots (7.1), the longest shoots, and the largest number of nodes (13.1) in cultures originating from nodal segments grown on MS medium with 1.0 mg L^−1^ of BA with the same concentration of indole-3-acetic acid (IAA) after 6 weeks. The number of nodes was increased by including glucose (Gluc) (Rahman et al., 2010). In propagation from shoot tips, the largest number of shoots and nodes and the longest shoot were on an MS medium with 0.5 mg L^−1^ of BA with 1.0 mg L^−1^ of IAA. New shoots were developed from one-node explants (average 1.94 ± 0.47) on medium with 0.1 mg L^−1^ of BA or kinetin in 4 weeks of *in vitro* propagation (Carloni et al., 2025). Gluc content in a medium influences axillary bud and callus formation; in two hop accessions from Czechia, 15 and 20 g L^−1^ of Gluc and 30 g L^−1^ of maltose produced the highest percentage of cultures with two shoots (Smýkalová et al., 2001). The best shoot proliferation in cv. ‘Cascade’ was reported on modified MS medium with 2.0 mg L^−1^ of meta-topolin and 0.5 mg L^−1^ of BA (9.48 ± 0.78 shoots/explant) (Clapa and Hârţa, 2021). A 96.6% shooting efficiency was reported on medium with 0.57 μM of IAA and 2.22 μM of BA, and 90.8% on medium with 2.85 μM of IAA and 29.52 μM of *N*^6^-(isopentenyl)adenine (2iP) (Lagos et al., 2022). However, even with a fair number of reports on hop micropropagation, there remains a gap in knowledge on PGRs promoting the development of a large number of shoots and nodes.

The US Department of Agriculture (USDA) ARS National Clonal Germplasm Repository in Corvallis (33707 Peoria Rd SW, Corvallis, OR 97333, USA) maintains a hop germplasm collection of 494 accessions (457 female and 37 male genotypes). To minimize the high cost of maintaining the germplasm in the field or in a screenhouse and to avoid exposing the collection to adverse field conditions, the germplasm was planned for long-term preservation in liquid nitrogen (i.e., cryogenic storage). Long-term preservation was performed at the ARS National Laboratory for Genetic Resources Preservation in Fort Collins, CO. To efficiently process a large number of accessions, micropropagation resulting in a large number of shoots and nodes in a relatively short time was required. The objective of our study was to test the effect of selected PGRs, such as BA, GA_3_, IAA, thidiazuron (TDZ), and *trans*-zeatin riboside (tZR), and the inclusion of Gluc in MS medium, on the development of the number of shoots, nodes, and the length of the main shoot in the micropropagation of three randomly selected accessions (PI 546055, PI 558687, and PI 617389) of *H. lupulus* L. var. *lupulus* during three micropropagation cycles, each lasting 4 weeks.

## Materials and methods

### Culture establishment

**Figure 1:**
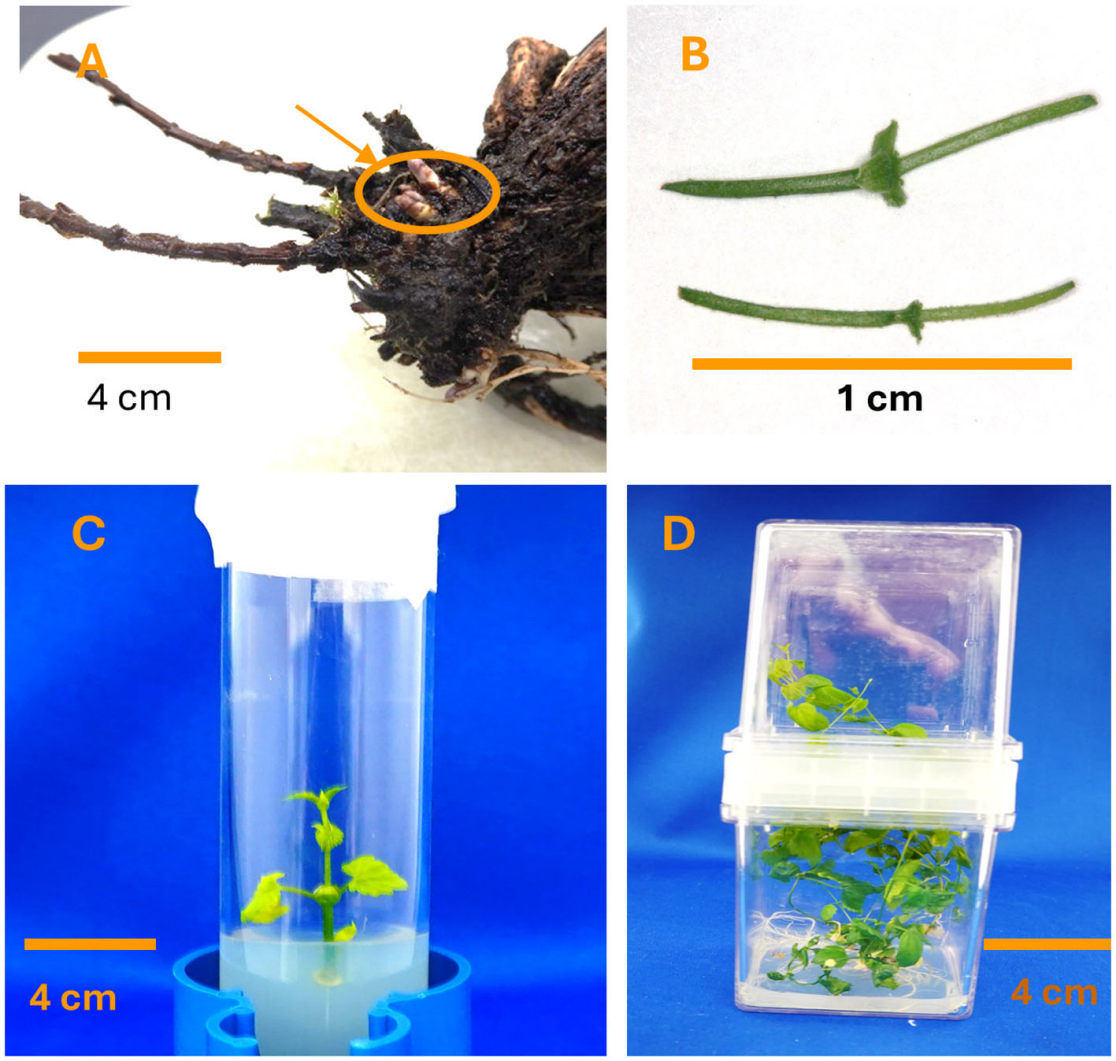
Images of *Humulus lupulus* L. var. *lupulus* material in a micropropagation experiment. **(A)** Rhizomes, **(B)** 1-cm nodal segments, **(C)** shoot in a test tube, and **(D)** shoots in coupled Magenta vessels.

Tissue cultures of three *H. lupulus* var. *lupulus* accessions (PI 546055, PI 558687, and PI 617389) were developed from rhizomes (**Figure 1A**) received from the USDA ARS National Clonal Germplasm Repository in Corvallis, OR. The rhizomes were grown in a chamber (25°C, 16 h photoperiod, 25 μmol·sec^−1^·m^−2^) in autoclaved peat moss for 3 weeks. Developed shoots were sterilized in a 10% commercial bleach (5.25% sodium hypochlorite) with 0.1 mL L^−1^ of Tween for 10 min and rinsed three times in sterile water under a laminar flow hood (LFH; Edge Gard, Sanford, ME). The shoots were cultured in test tubes with 5 mL of ½ strength liquid MS medium (Murashige and Skoog, 1962) with vitamins (M519, PhytoTech Labs, Lenexa, KS, USA) for 4 weeks; contaminated cultures were discarded. Clean cultures were multiplied on solid propagation medium [MS with vitamins (M519), 0.1 mg L^−1^ of BA (B3408, Millipore Sigma, Burlington, MA, USA), 0.2 g L^−1^ of Sequestrene 138 (E349, PhytoTech Labs), 20 g L^−1^ of glucose (G8270, Millipore Sigma), 3 g L^−1^ of agar (A1296, Sigma-Aldrich, St. Louis, MO, USA), and 1.5 g L^−1^ of gelrite (A7002, Sigma), with pH 5.4 adjusted with KOH] dispensed in test tubes in propagation cycles 1 and 2 and in Magenta GA7 vessels (Magenta Corporation, Chicago, IL, USA) in cycle 3 and autoclaved at 121°C and 20 psi for 20 min. After autoclaving, the pH was 5.0. Cultures were transferred to fresh propagation medium every 4 weeks. The cultures were cultivated in a growth chamber under the same conditions as during rhizome growth.

### Experimental setup

Once enough nodes were developed, an experiment on propagation medium with selected treatments of PGRs and on medium with Gluc without PGRs was initiated. One-centimeter nodal segments (**Figure 1B**) with two opposite buds were placed in test tubes (ø 24 × 150 mm) with 16 mL of propagation medium with or without PGRs [14 treatments and a control (standard propagation medium used in our laboratory) (**Table 1**)]. For each accession and treatment, three replications with three test tubes/replication were initiated and propagated for three propagation cycles: in cycles 1 and 2 in three test tubes (one shoot/tube) (**Figure 1C**) and in cycle 3 in Magenta GA7 vessels (double vessels connected with a coupler, nine cultures/box) (**Figure 1D**) with the same treatments. The double vessels were used to provide space for shoot development. Each propagation cycle was 4 weeks long. In consecutive cycles, cultures were transferred to the medium with the same treatment.

**Table 1:** Treatments of plant growth regulators and glucose treatments tested in a micropropagation experiment of three accessions (PI 546055, PI 558687, and PI 617389) of *Humulus lupulus* L. var. *lupulus*.

Treatment (PGR in MS medium)	Concentration (mg L^−1^)
Control (BA + Gluc)	0.1 + 20 g L^−1^
GA_3_	0.05
BA + GA_3_	0.01 + 0.025
BA + GA_3_	0.01 + 0.05
BA	0.5
TDZ	0.1
TDZ	0.2
TDZ	0.5
tZR + IAA	0.1 + 0.01
tZR + IAA	0.2 + 0.01
tZR + IAA	0.5 + 0.01
tZR	3.0
Gluc g L^−1^	15 g L^−1^
Gluc g L^−1^	30 g L^−1^
Gluc g L^−1^	45 g L^−1^

PGR, plant growth regulator; MS, Murashige and Skoog; BA, benzyladenine; Gluc, glucose; GA_3_, gibberellic acid; TDZ, thidiazuron; tZR, *trans*-zeatin riboside; IAA, indole-3-acetic acid.

### Data collection and statistical evaluation

Data on the number of shoots, nodes, and the length of the main shoot (cm) were collected at the end of each propagation cycle (each cycle had three replications, three test tubes/replication in cycles 1 and 2, and one Magenta box/replication in cycle 3) for each treatment. Data were analyzed using a Fit Model function in JMP 18 (Statistical Discover LLC software, a subsidiary of SAS Institute, Cary, NC, USA). Graphs were constructed using a Graph Builder in the same JMP18 software.

## Results and discussion

The characteristics evaluated differed between accessions, propagation cycles, and treatments.

### Number of shoots

**Figure 2:**
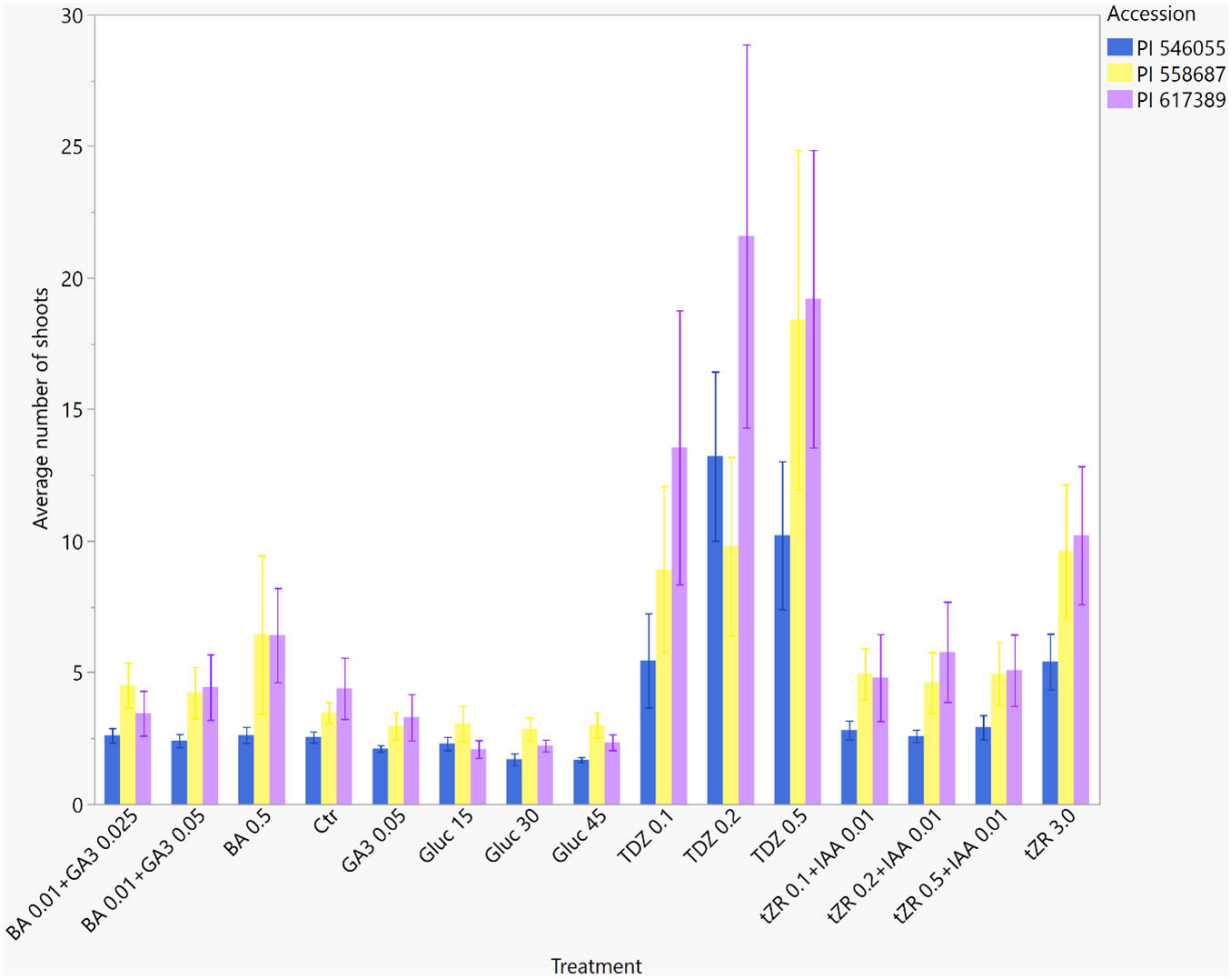
Average number of shoots in three *Humulus lupulus* L. var. *lupulus* accessions (PI 546055, PI 558687, and PI 617389) propagated *in vitro* in 15 treatments for three cycles (total 12 weeks).

The largest number of shoots (including the main and lateral shoots) was observed in TDZ 0.5 and 0.2 mg L^−1^ treatments (15.9 ± 1.34 and 14.9 ± 1.34, respectively). The lowest number of shoots was in 0.05 mg L^−1^ of GA_3_ (2.8 ± 1.34) and all treatments with Gluc (**Table 2**). Shoot number was not significantly different between TDZ 0.1, tZR 3.0, and TDZ 0.2 mg L^−1^. In the other treatments (BA 0.5, tZR with IAA, and the control), the shoot number was not significantly different (from 5.2 ± 1.34 to 3.5 ± 1.34). The lowest average number of shoots was observed in GA_3_ and glucose treatments without PGRs. The shoot number in PI 617389 in cycle 3 was significantly higher than in all other accessions and cycles (**Table 3**). The number of shoots in the control treatment routinely used in our laboratory for hop micropropagation was significantly lower than the number of shoots developed in TDZ treatments and on 3.0 mg L^−1^ of tZR. The number of shoots for each accession in each treatment is shown in **Figure 2**.

**Table 2:** Average number of shoots in three *Humulus lupulus* L. var. *lupulus* accessions (PI 546055, PI 558687, and PI 617389) cultivated *in vitro* in 15 treatments during three propagation cycles (total 12 weeks).

Treatment	mg L^−1^	Least sq. mean
TDZ	0.5	15.9 A
TDZ	0.2	14.9 AB
TDZ	0.1	9.3 BC
tZR	3.0	8.4 BCD
BA	0.5	5.2 CD
tZR + IAA	0.2 + 0.01	4.3 CD
tZR + IAA	0.5 + 0.01	4.3 CD
tZR + IAA	0.1 + 0.01	4.2 CD
BA + GA_3_	0.01 + 0.05	3.7 CD
BA + GA_3_	0.01 + 0.025	3.5 CD
Control	0.1 + 20 g L^−1^	3.5 CD
GA_3_	0.05	2.8 D
Gluc	15 g L^−1^	2.5 D
Gluc	45 g L^−1^	2.3 D
Gluc	30 g L^−1^	2.3 D

p < 0.0001. SE ± 1.34. Least sq. mean differences with Tukey's HSD. Alpha 0.05.

TDZ, thidiazuron; tZR, *trans*-zeatin riboside; BA, benzyladenine; IAA, indole-3-acetic acid; GA_3_, gibberellic acid; Gluc, glucose.

**Table 3:** Average number of shoots in each *Humulus lupulus* L. var. *lupulus* accession cultivated *in vitro* in 15 treatments for three propagation cycles (total 12 weeks).

Accession	Cycle	Least sq. mean
PI 617389	3	15.1 A
PI 558687	3	12.4 A
PI 546055	3	6.5 B
PI 617389	2	4.7 BC
PI 558687	2	3.9 BC
PI 546055	2	3.4 BC
PI 546055	1	2.1 BC
PI 558687	1	2.1 BC
PI 617389	1	2.0 C

p < 0.0.001. SE ± 1.0. Least sq. mean differences with Tukey's HSD. Alpha 0.05.

**Figure 3:**
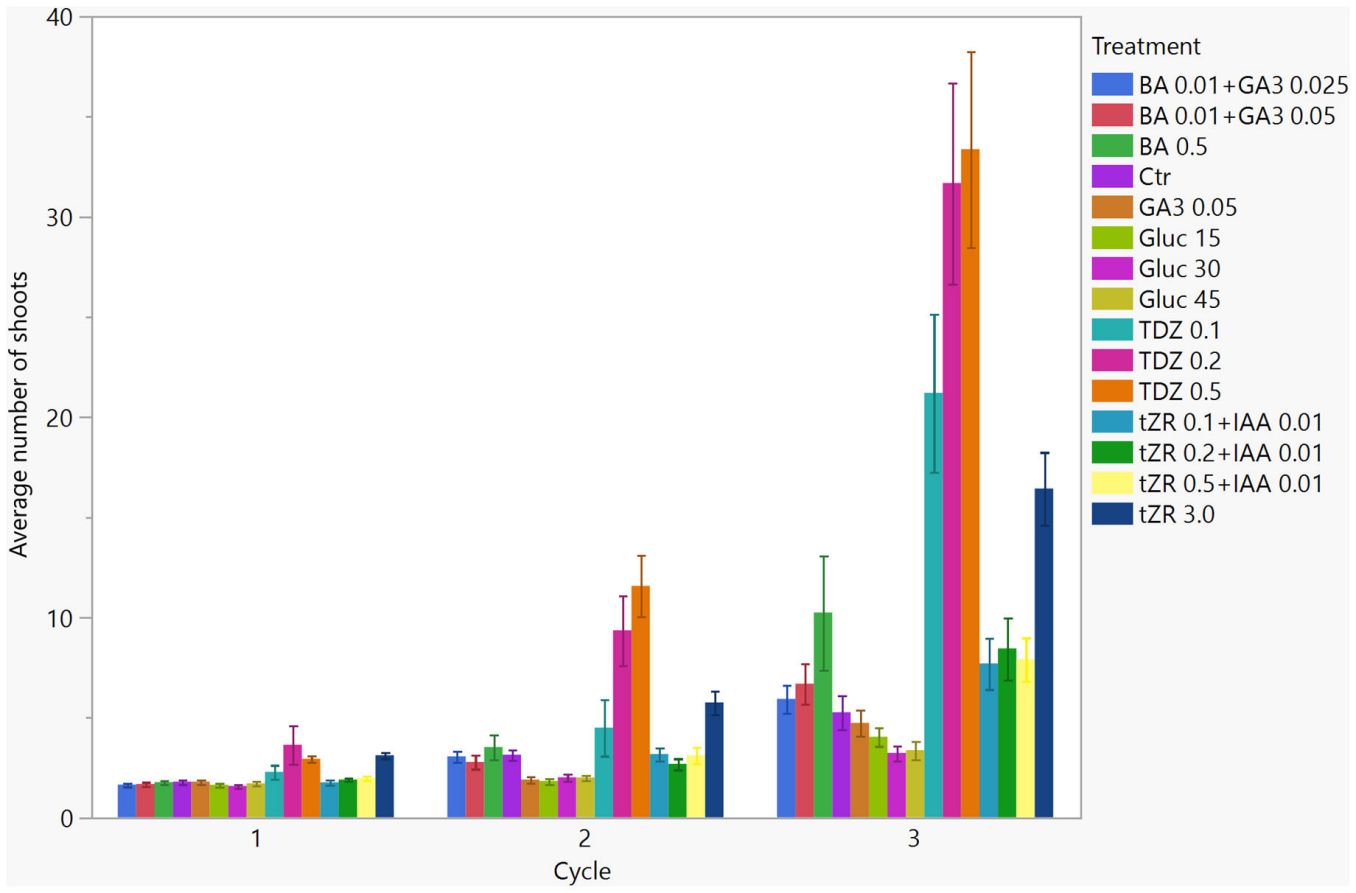
Average number of shoots of three *Humulus lupulus* L var. *lupulus* accessions (PI 546055, PI 558687, and PI 617389) in each *in vitro* propagation cycle (total 12 weeks) in 15 treatments.

In 2018, Machado et al. reported the largest number of shoots for the ‘Columbus’ cultivar on medium with 0.2189 mg L^−1^ TDZ. Iacuzzi et al. (2023) observed the highest number of shoots on medium with 2 mL L^−1^ of BA (concentration in the BA solution not known, assumed 1 mg/mL). Our treatment of 0.5 mg L^−1^ of BA was lower than in the Iacuzzi et al. report and resulted in a lower number of shoots. BA is the most frequently used PGR in plant propagation. Lu (1993) and MaChado et al. (2018) reported the largest shoot number on medium with TDZ, but the effect of TDZ depended on the concentration used (Dewir et al., 2018). Large shoot numbers generate more nodal segments for micropropagation than a lower number of shoots. Most shoots were developed in the third propagation cycle (**Figure 3**); the third propagation was performed in larger tissue culture vessels (double Magenta GA7 vessels) rather than test tubes, as in cycles 1 and 2, but utilizing the same treatment across the cycles. Larger vessels (Magenta boxes) were a good option for hop micropropagation because they supported vigorous shoot development; however, genotype may have played a role, too. In each successive propagation cycle, the number of shoots increased significantly and was substantially different between the three accessions; the highest was in PI 617389 (15.1 ± 1.0) and PI 558687 (12.4 ± 1.0) in cycle 3. Overall, the shoot number between the three accessions had low variability (**Table 4**). In *Arabidopsis thaliana* (L.) Heynh. and *Populus* L., GA_3_ inhibited the development of axillary buds but promoted axillary buds’ growth in beans, citrus, and hop, suggesting that a development mechanism controlled by GA_3_ is complicated in hop cultivars and is different between hop cultivars (Hirakawa and Tanno, 2022).

**Table 4:** Average number of shoots for three accessions of *Humulus lupulus* L. var. *lupulus* cultivated *in vitro* for three micropropagation cycles (total 12 weeks).

Accession	Least sq. mean
PI 617389	7.3 A
PI 558687	6.1 AB
PI 546055	4.0 B

p = 0038. SE ± 0.69. Least sq. mean differences with Tukey's HSD. Alpha 0.05.

### Number of nodes

**Figure 4:**
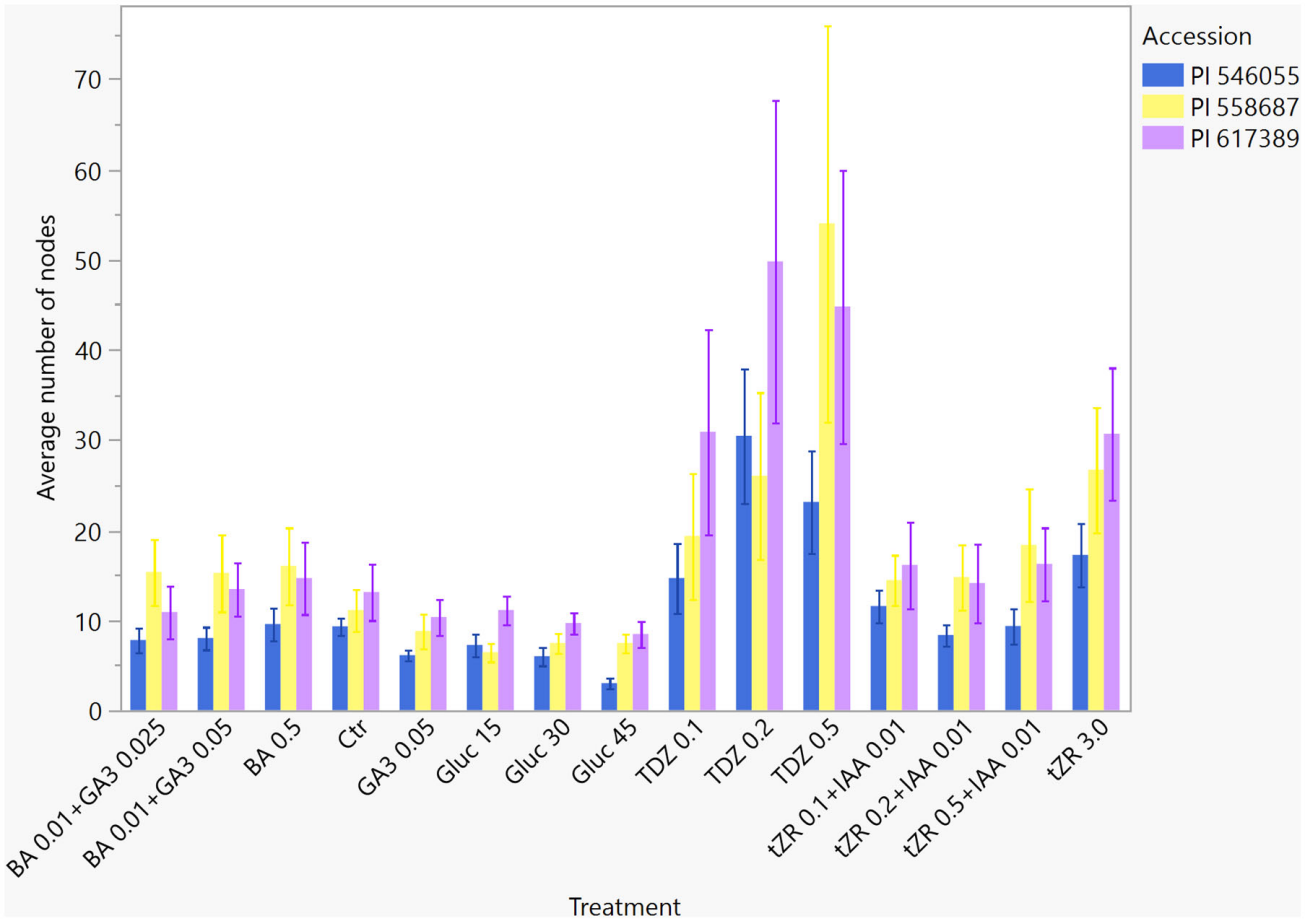
Average number of nodes of three *Humulus lupulus* L. var. *lupulus* accessions (PI 546055, PI 558687, and PI 617389) propagated *in vitro* in 15 treatments for three cycles (total 12 weeks).

The number of nodes affects the micropropagation rate at a later time and overall depends on the number of shoots. The trend for average node number in each treatment was similar to the number of shoots, with the most in TDZ 0.5 (40.0 ± 3.6), TDZ 0.2 (36.4 ± 3.6), and tZR 3.0 mg L^−1^ (24.9 ± 3.6) (**Table 5**). The lowest node number was in the 45 g L^−1^ glucose treatment (6.3 ± 3.6); in all other treatments, the differences in the node numbers were not significant. The average number of nodes was significantly greater in cycle 3 for PI 617389 (39.8 ± 2.54) and PI 558687 (36.0 ± 2.54) (**Table 6**) than the number in PI 546055 (18.6 ± 2.54). In the other propagation cycles, the lowest node number was in cycle 1 for all three accessions tested. An interaction between accessions and treatment was at p = 0.0006, and cycle and treatments at p < 0.0001. In a hop micropropagation experiment, 0.02 mg L^−1^ of IAA combined with 1.0 mg L^−1^ of BA contributed to the largest number of nodal segments in ‘Chinook’ and ‘Columbus’ hop cultivars (De-Souza et al., 2021a). In our experiment, 0.01 mg L^−1^ of IAA in combination with 0.5 and 0.1 mg L^−1^ of tZR did not support a large number of nodes (**Table 5**). **Figure 4** shows the number of nodes developed in the three-hop accessions cultivated in the treatments tested.

**Table 5:** Average number of nodes of three *Humulus lupulus* L. var. *lupulus* accessions (PI 546055, PI 558687, and PI 617389) cultivated *in vitro* in 15 treatments for three propagation cycles (total 12 weeks).

Treatment	mg L^−1^	Least sq. mean
TDZ	0.5	40.6 A
TDZ	0.2	36.4 AB
tZR	3.0	24.9 ABC
TDZ	0.1	21.6 BCD
tZR + IAA	0.5 + 0.01	14.6 CD
tZR + IAA	0.1 + 0.01	14.0 CD
BA	0.5	13.4 CD
tZR + IAA	0.2 + 0.01	12.4 CD
BA + GA_0_	0.01 + 0.05	12.2 CD
BA + GA_3_	0.01 + 0.025	11.3 CD
Control	0.1 + 20 g L^−1^	11.1 CD
GA_3_	0.05	8.4 CD
Gluc	15 g L^−1^	8.3 CD
Gluc	30 g L^−1^	7.7 CD
Gluc	45 g L^−1^	6.3 D

p < 0.0001. SE ± 3.6. Least sq. mean differences with Tukey's HSD. Alpha 0.05.

TDZ, thidiazuron; tZR, *trans*-zeatin riboside; IAA, indole-3-acetic acid; GA_3_, gibberellic acid; Gluc, glucose.

**Table 6:** Average number of nodes in each accession of *Humulus lupulus* L var. *lupulus* propagated *in vitro* in 15 treatments for three propagation cycles (total 12 weeks).

Accession	Cycle	Least sq. mean
PI 617389	3	39.8 A
PI 558687	3	36.0 A
PI 546055	3	18.6 B
PI 617389	2	13.4 BC
PI 558687	2	11.6 BC
PI 546055	2	11.0 BC
PI 617389	1	5.6 C
PI 558687	1	4.6 C
PI 546055	1	4.6 C

p < 0.0001. SE ± 2.5. Least sq. mean differences with Tukey's HSD. Alpha 0.05.

### Length of main shoot

**Figure 5:**
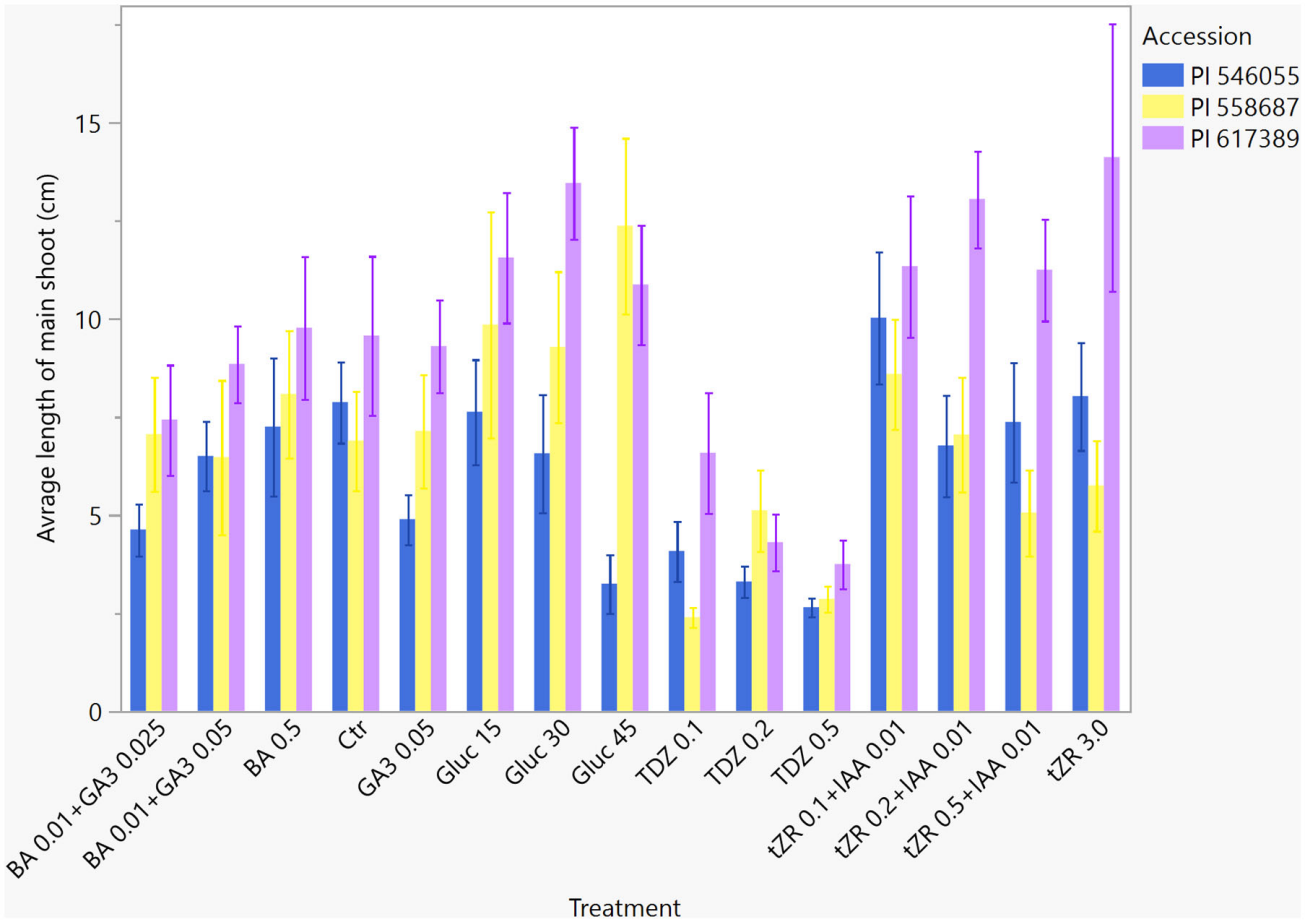
Average length of main shoot (cm) for three accessions of *Humulus lupulus* L. var. *lupulus* accessions (PI 546055, PI 558687, and PI 617389) cultivated *in vitro* in 15 treatments for three propagation cycles (total 12 weeks).

The average length of the main shots was not significantly different for the majority of the treatments tested. The main shoot in the TDZ 0.5 mg L^−1^ treatment was significantly shorter (**Table 7**). After the third propagation cycle (total of 12 weeks of *in vitro* cultivation), the longest average main shoot (cm) was in PI 617389 (13.5 ± 0.56) and PI 558687 (11.1 ± 0.56). The shortest main shots were PI 546055 and PI 558687 in cycle 1. In cycle 2, the average length of the main shoot in PI 558687 and PI 546055 was not significantly different than that in PI 546055 in cycle 3 (p = 0.1367) (**Table 8**). **Figure 5** shows the length of the main shoot for each accession and each treatment. Across the three propagation cycles, PI 617389 and PI 558687 had significantly longer main shoots than PI 546055, suggesting that the characteristic may be genotype-dependent. Longer shoots provide more nodal segments for micropropagation than shorter ones; hence, accessions that develop long shoots may be propagated faster than accessions with shorter shoots.

**Table 7:** Average length of main shoot (cm) in three *Humulus lupulus* L. var. *lupulus* accessions (PI 546055, PI 558687, and PI 617389) cultivated *in vitro* in 15 treatments for three propagation cycles (total 12 weeks).

Treatment	mg L^−1^	Least sq. mean
tZR + IAA	0.1 + 0.01	10.0 A
Gluc	30 g L^−1^	9.8 A
Gluc	15 g L^−1^	9.7 A
tZR	3.0	9.3 A
tZR + IAA	0.2 + 0.01	9.0 A
Gluc	45 g L^−1^	8.8 A
BA	0.5	8.4 AB
Control	0.1 + 20 g L^−1^	8.1 ABC
tZR + IAA	0.5 + 0.01	7.9 ABC
BA + GA_3_	0.01 + 0.05	7.3 ABC
GA_3_	0.05	7.1 ABCD
BA + GA_3_	0.01 + 0.025	6.4 ABCD
TDZ	0.1	4.3 BCD
TDZ	0.2	4.2 CD
TDZ	0.5	3.1 D

p < 0.0001. SE ± 0.92. Least sq. mean differences with Tukey's HSD. Alpha 0.05.

tZR, *trans*-zeatin riboside; IAA, indole-3-acetic acid; Gluc, glucose; BA, benzyladenine; GA_3_, gibberellic acid; TDZ, thidiazuron.

**Table 8:** Average length of main shoot (cm) for each tested accession of *Humulus lupulus* L. var. *lupulus* cultivated *in vitro* for three propagation cycles.

Accession	Cycle	Least sq. mean
PI 617389	3	13.5 A
PI 558687	3	11.1 AB
PI 617398	2	10.1 B
PI 546055	3	8.7 BC
PI 558687	2	7.2 CD
PI 546055	2	6.7 CD
PI 617389	1	5.4 D
PI 546055	1	2.8 E
PI 558687	1	2.5 E

p = 0.1367. SE ± 0.56. Least sq. mean differences with Tukey's HSD. Alpha 0.05.

In a hop micropropagation experiment, the shoot length was affected by the concentration of a PGR and less by the PGR type (Bychkova et al., 2024). The effect of TDZ depends on its concentration and varies by plant species or genotypes within a species (Deepa et al., 2018; Roy et al., 2001). Our data support the benefits of consecutive micropropagation on media with the same PGR and using larger culture vessels instead of test tubes. Long main shoots and a high number of shoots and nodes contribute to an increased number of plantlets in micropropagation. Although there was a significant difference in node number between cycles and the tested accessions, the PGR treatments were not of importance for the node number in the three tested hop genotypes, but 0.5 and 0.2 mg L^−1^ of TDZ and 3.0 mg L^−1^ of tZR showed the highest average number of nodes. PI 546055 cultures propagated in TDZ treatments showed a physiological disorder of hyperhydricity, with 33% plantlets of cycle 1 (on 0.1 mg L^−1^), 66% plantlets (on 0.2 mg L^−1^) of cycle 2, and 100% plantlets (on 0.5 mg L^−1^) of cycle 3; thus, TDZ should be used carefully in hop micropropagation when the genotype reaction to TDZ is not known. Hyperhydricity decreases the efficacy of clonal micropropagation. The disorder may be caused by oxidative stress, hormonal and mineral composition, exogenous supplements, impaired nitrogen metabolism, and insufficient gas exchange, and plant genotypes may react differently to different factors (Lelis et al., 2025; Pasternak and Steinmacher, 2025; Polivanova and Bedarev, 2022; Singh, 2024). In our experiment, the three genotypes were exposed to the same cultivation conditions and TDZ concentrations, but only PI 546055 showed symptoms of hyperhydricity; hence, we assume that the genotype of this accession was sensitive to TDZ. TDZ may also impair shoot elongation and shoot development (Pai and Desai, 2018; Lelis et al., 2025).

Other treatments, in addition to TDZ that resulted in a large number of nodes, may be applicable in hop micropropagation (e.g., 3.0 mg L^−1^ of tZR), but their application may be limited by high cost and challenges in production, regulation, and their inhibitory effects on secondary root development (Kuroha et al., 2002). The effect of treatments on the number of nodes and shoot length depends on plant genotype (Bychkova et al., 2024). The observed differences in this experiment also demonstrate genotype differences. Long main shoots and a high number of shoots and nodes contribute to an increased number of plantlets in micropropagation. High sprouting efficiency (96.6%) was reported for a medium with 0.57 μM of IAA and 2.22 μM of BA and for 29.52 μM of 2iP (Lagos et al., 2022). Experiments of De-Souza et al. (2021b) and Peredo et al. (2009) showed no phenotypic changes in hop plantlets propagated *in vitro* for 3 months. In our experiment, no phenotypic changes were observed during the 12 weeks of *in vitro* cultivation, except for a physiological disorder of hyperhydricity in TDZ treatments. Our experiment identified a more productive treatment than the one currently used for hop micropropagation (i.e., control).

### Selection of MS medium with glucose

In this experiment, MS (Murashige and Skoog, 1962) medium with an N:P ratio of 40:1 was used. Recent publications of Pasternak and Steinmacher (2024, 2025) have questioned the MS medium application for mass multiplication and have suggested that this medium is applicable to a limited number of species and may cause hyperhydricity. We observed hyperhydricity only in one accession (PI 546055) out of the three tested; most likely, in this accession, the physiological disorder was genotype-dependent. In Hirakawa and Tanno’s (2022) experiment, MS media with 20 g L^−1^ of glucose supported the axillary bud development of hop tissue culture, and Reed et al. (2003) successfully used MS with 20 g L^−1^ of glucose for establishing cultures of 70 hop genotypes for cold storage (4°C). In *in vivo* plants, glucose is the most frequently used carbon source. In the media, glucose may be unstable and degrade to lactic acid; this degradation product may be toxic to cells and other tissues (Pasternak and Steinmacher, 2024). The level of glucose degradation products (e.g., increased hydrogen peroxidase activity and carboxymethyl-lysine) was used to determine the level of autoxidative reactions during protein glycation induced by glucose (Avila et al., 2017). Heating 40% glucose solution at 55°C for 5 weeks decreased the pH of the solution and increased the concentration of formic acid, which is thought to be a glucose degradant (Moore et al., 2020). A decrease in pH after media autoclaving was reported by Skirvin et al. (1986); the authors recommended measuring pH before and after autoclaving, which was performed in our experiment. Autoclaving parenteral solution with glucose at 121°C for 15 min resulted in very low levels of the glucose degradation products such as 3-deoxyglucosone and 5-hydroxymethylfurfural (Leitzen et al., 2021). According to Witkowski and Jörres (2000), glucose may degrade spontaneously with time and during sterilization at high temperatures. Glucose degradation products (e.g., α-dicarbonyl, 3-deoxyhexosulose, and methylglyoxal) may result in isomerization products and smaller carbohydrates (Hollnagel and Kroh, 2000) and intermediate products (Gobert and Glomb, 2009). Glucose is considered a universal carbon source in plants; it is also a signaling molecule (Shah et al., 2019; Siddiqui et al., 2020). In plant tissue culture media, glucose is relatively stable; however, it may degrade under high temperatures or low pH into glyoxal, methylglyoxal, glucosone, and other products. Leitzen et al. (2021) and Kjellstrand et al. (2004) recommended storing media with glucose below 20°C. The media used in our experiment were prepared 1–2 days before culture transfer and stored at 12°C. The ratio of glucose degradation in media may be highly variable and change during storage and/or by usage of plant cultures (Leitzen et al., 2021; Witkowski and Jörres, 2000). In *Brassica napus* L. culture medium, glucose content was stable during the entire culture period (Ślesak et al., 2004). In *Amaranthus caudatus* L. roots, a 59-fold increase in gluconic acid was thought to be a result of direct glucose oxidation (Osmolovskaya et al., 2025). In our experiment, the observed lower pH in the media after autoclaving may be due to glucose degradation products. The type and level of the potential degeneration products may require further investigation.

## Conclusion

The number of shoots and nodes and the length of the main shoot were genotype-dependent; the three characteristics were the largest in PI 617389, followed by PI 558687. The highest numbers of shoots and nodes were observed on 0.5 and 0.2 mg L^−1^ of TDZ and in 3.0 mg L^−1^ tZR treatments; however, cultures of PI 546055 manifested a physiological disorder of hyperhydricity in all TDZ concentrations. This plant growth regulator should be used carefully in *in vitro* hop propagation. TDZ also caused shorter shoots or no shoot growth, which is a disadvantage where longer shoots are desired. tZR in a concentration of 3.0 mg L^−1^ may be an effective plant growth regulator in hop micropropagation, aiming for a large number of clonal propagules. In this treatment, the evaluated traits, such as the number of shoots, the number of nodes, and the length of the main shoot, were larger than those in the control treatment routinely used in hop micropropagation in our laboratory. Consecutive *in vitro* transfers to the same PGR and propagation in large culture vessels (two coupled Magenta G7 vessels) contributed to a larger number of shoots and nodes and longer shoots in comparison to other plant growth regulators tested. Both PGRs, TDZ and tZR, may be applied in routine hop micropropagation for commercial and research purposes where a large number of propagules are required, but in the case of TDZ, the tendency of the material to develop hyperhydricity has to be established before mass propagation. tZR is one of the most effective cytokinins in plant growth and development; it may cause hyperhydricity, but to a negligible extent. The physiological disorder was not observed in the hop genotypes tested on media with the phytohormone.

## Data Availability

The raw data supporting the conclusions of this article will be made available by the authors, without undue reservation.
